# LncRNA and mRNA expression characteristic and bioinformatic analysis in anemic diabetic foot ulcers

**DOI:** 10.3389/fgene.2025.1603315

**Published:** 2025-09-29

**Authors:** Jiayu Lin, Jingying Wang, Bo Liang

**Affiliations:** Department of Endocrinology, The Second Affiliated Hospital of Fujian Medical University, Quanzhou, Fujian, China

**Keywords:** bioinformatic analysis, diabetic foot ulcers, long non-coding RNAs, anemia, iron metabolism dysregulation, chronic inflammation

## Abstract

**Background:**

Diabetic foot ulcers (DFUs) are severe complications often accompanied by hemoglobin deficiency. While transcriptomic alterations are implicated in DFU pathogenesis, the roles of long non-coding RNAs (lncRNAs) in linking hemoglobin anomalies to iron-inflammatory dysregulation remain unclear.

**Objective:**

To identify DFU-specific lncRNA-mRNA co-expression networks associated with iron metabolism and inflammation, and evaluate their diagnostic potential.

**Methods:**

RNA sequencing (Illumina NovaSeq 6000) was performed on skin tissues from 5 patients with type 2 diabetes and DFUs and 3 non-diabetic controls. Differentially expressed lncRNAs and mRNAs were identified using DESeq2. Functional pathway enrichment was assessed via Gene Set Enrichment Analysis (GSEA). Correlation networks integrating expression data were constructed to map lncRNA-mRNA interactions.

**Results:**

Analysis revealed 21 differentially expressed lncRNAs (1 upregulated, 20 downregulated) and 368 differentially expressed mRNAs (139 upregulated, 229 downregulated) in DFU tissues. GSEA confirmed significant enrichment of hemoglobin-related pathways, including iron uptake/transport, oxidative stress, and neutrophil degranulation. A co-expression network integrating these findings mapped interactions between 21 lncRNAs and 21 mRNAs. Key lncRNAs showed strong correlations with mRNAs involved in iron dysregulation and chronic inflammation, suggesting their contribution to DFU pathology.

**Conclusion:**

Our analysis of anemia in DFU identifies distinct lncRNA-mRNA co-expression networks linked to hemoglobin deficiency. Key lncRNAs correlate strongly with mRNAs involved in perturbed iron handling and sustained inflammation. This suggests that dysregulation within these networks significantly contributes to the pathology of DFU with concurrent anemia, highlighting potential regulatory roles for these lncRNAs and offering avenues for diagnostic development.

## Introduction

Diabetes mellitus (DM) is a group of metabolic disorders characterized by chronic hyperglycemia, resulting from either insufficient insulin secretion or ineffective insulin action. These impairments are influenced by both genetic and environmental factors (American Diabetes Association, 2014). DM is one of the most prevalent metabolic conditions globally, especially in countries like India, China, and the United States. As of 2013, it affected approximately 382 million individuals worldwide (Zimmet et al., 2014), and projections indicate that this number will rise to around 592 million by 2035 (Kharroubi and Darwish, 2015). Among the various complications of DM, diabetic foot ulcers (DFUs) are among the most severe, affecting 15%–25% of individuals with diabetes (Lipsky et al., 2012). Recent studies emphasize the significant burden DFUs place on individuals and healthcare systems due to their high morbidity and frequent hospitalizations (Liu et al., 2023). While neuropathy, ischemia, and infection are established contributors to DFU pathogenesis (Aumiller and Dollahite, 2015), emerging evidence highlights anemia as a critical yet understudied comorbidity in DFU patients. Anemia prevalence in diabetic populations exceeds 30% and correlates with impaired wound healing and ulcer severity (Faghir-Ganji et al., 2024; Humar et al., 2022), suggesting its direct involvement in DFU progression.

Central to this association is the dysregulation of iron homeostasis and chronic inflammation. Iron deficiency disrupts oxygen delivery and collagen synthesis, while iron overload (via impaired recycling) fuels oxidative stress and inflammatory cascades (Wright et al., 2014). In DFUs, aberrant iron handling manifests as elevated hepcidin (a key iron regulator), suppressed erythropoiesis, and persistent neutrophil activation–processes implicated in tissue damage and impaired repair (Alamo et al., 2016). Despite this mechanistic link, the transcriptional drivers connecting hemoglobin anomalies to iron-inflammatory imbalance remain poorly defined.

Long non-coding RNAs (lncRNAs), a subclass of ncRNAs, are typically longer than 200 nucleotides and do not encode proteins (Bus et al., 2024). LncRNAs exert regulatory effects on protein-coding genes through epigenetic, transcriptional, and post-transcriptional mechanisms (Ferrer and Dimitrova, 2024). Long non-coding RNAs (lncRNAs) have emerged as pivotal regulators of metabolic and inflammatory pathways in diabetes (Kuai et al., 2022). Notably, lncRNAs modulate iron metabolism (e.g., LncRNA H19 suppresses hepcidin expression) (Wang et al., 2021) and inflammation (e.g., LncRNA MALAT1 regulates NF-κB signaling) (Hussain et al., 2023). However, their roles in integrating hemoglobin deficiency with iron-inflammatory dysregulation in DFUs are entirely unexplored. Current studies on DFU transcriptomics focus predominantly on protein-coding genes (Su et al., 2024), leaving a critical gap in understanding lncRNA-mediated regulatory networks specific to anemia-associated DFU pathology.

To address this, we performed RNA sequencing of DFU tissues from patients with concurrent anemia and non-diabetic controls. Our study aims to identify DFU-specific lncRNA-mRNA co-expression networks linked to hemoglobin-related pathways (iron uptake/transport, oxidative stress, neutrophil activation), offering novel targets for biomarker development and targeted interventions.

## Materials and methods

Skin tissue samples in this study were obtained from two groups: the non-diabetic control (Group A) and the diabetic foot group (Group B). The samples were collected from five DFU patients and three patients with acute wounds who met the inclusion criteria and were treated at the Second Affiliated Hospital of Fujian Medical University between January 2023 and June 2023. The DFU group consisted of three male and two female patients, while the acute wound group included two male and one female patient. These samples were used for subsequent experimental research. All participants provided informed consent and were enrolled according to established criteria. All experiments were performed in accordance with relevant guidelines and regulations in the method section. All experimental procedures were approved by the Ethics Committee of the Second Affiliated Hospital of Fujian Medical University.

Upon admission, skin tissue samples were collected from all participants by trained medical professionals. Relevant demographic data, medical history, and other clinical information were also recorded for each participant.

The inclusion criteria for patients with DFU were those who met the diagnostic criteria for diabetic foot ulcers (Kuai et al., 2022). Exclusion criteria for DFU patients included individuals with any coexisting wound infection, encompassing local infections confined to the ulcer site as well as infections that had spread to become systemic.

For normal skin tissue patients, the inclusion criteria were individuals with wounds who had post-surgical skin tissue free from infection. The exclusion criteria included individuals with sepsis, autoimmune diseases, malignant tumors, or other significant systemic diseases.

### Sample collection, RNA extraction, and sequencing

Tissue samples from diabetic foot ulcers and non-diabetic control wounds were collected, with surface liquid removed, and immediately placed into enzyme-free cryogenic vials. The samples were snap-frozen in liquid nitrogen and subsequently stored at −80 °C until further processing. Total RNA was isolated from approximately 30 mg of homogenized tissue using the HiPure Total RNA Mini Kit (Magen Biotechnology, Guangzhou, China, Cat. No. R4130-03) following the manufacturer’s protocol. RNA concentration and purity were determined using a NanoDrop spectrophotometer (Thermo Fisher Scientific, Waltham, MA, United States of America). RNA integrity was assessed using an Agilent 2100 Bioanalyzer (Agilent Technologies, Santa Clara, CA, United States). RNA quality was examined by gel electrophoresis and with Qubit. Only samples with an RNA Integrity Number (RIN) ≥ 8.0, OD260/280 ≥ 1.8, and OD260/230 ≥ 1.0 were used for subsequent library preparation.

Sequencing libraries were constructed from 1 μg of high-quality total RNA per sample using the VAHTS Total RNA-seq (H/M/R) Library Prep Kit for Illumina (Vazyme Biotech, Nanjing, China, Cat. No. NR603). This kit utilizes a probe-based method for ribosomal RNA (rRNA) depletion and includes all steps for cDNA synthesis, end repair, adapter ligation, and PCR amplification. The final libraries were quantified using a Qubit fluorometer (Thermo Fisher Scientific) and their size distribution was confirmed using an Agilent 2100 Bioanalyzer. The qualified libraries were sequenced on an Illumina NovaSeq 6000 platform (Illumina, San Diego, CA, United States) with a paired-end 150 bp (PE150) strategy. The sequencing depth averaged 12.8 Gb per sample, with an average Q30 score of 95.0%.

### Bioinformatic analysis

Raw paired-end reads were processed through a standardized pipeline. Adapter sequences and low-quality bases were trimmed using Skewer (v0.2.2). The resulting clean reads were aligned to the human reference genome GRCh38.p13 and its corresponding annotation (Homo_sapiens.GRCh38.109.gtf) from Ensembl using STAR (v2.7.10a) in two-pass mode. A reference-guided transcriptome assembly was then performed for each sample using StringTie (v2.2.1), and the resulting transcripts were merged across all samples to create a unified transcriptome using StringTie’s--merge function. Transcript abundance was quantified against this merged assembly using RSEM (v1.3.3), and expression levels were normalized and reported as Transcripts Per Million (TPM).

### Identification and classification of lncRNAs

Transcripts were classified to distinguish lncRNAs from mRNAs. Known lncRNAs were directly identified from the Ensembl annotation (biotypes: lncRNA, antisense). For novel transcripts, a multi-step filtering strategy was applied. First, assembled transcripts were compared to the reference annotation using gffcompare, and unknown intergenic, intronic, and antisense transcripts were classified as preliminary lncRNA candidates. Second, candidates that were single-exon or shorter than 200 nucleotides were filtered out to remove potential artifacts. Finally, the coding potential of the remaining transcripts was assessed using four independent tools: PLEK, CPAT, CNCI, and CPC2. To maximize confidence, only transcripts predicted as non-coding by all four tools were retained as novel lncRNAs. All protein-coding transcripts were classified as mRNAs for subsequent analysis.

### Differential expression and enrichment analysis

Differential expression analysis between the DFU and non-diabetic control groups was performed using DESeq2 (v1.10.1). Differentially expressed lncRNAs (DE-lncRNAs) and mRNAs (DE-mRNAs) were identified based on |log2 fold change| ≥ 0.5 and a Benjamini–Hochberg adjusted P-value <0.05. Volcano plots, generated with ggplot2 in R, provided a visual representation of the results, illustrating both the fold change and significance levels of gene expression. Gene Set Enrichment Analysis (GSEA; MSigDB v7.5.1) was applied to DE-mRNAs using pre-ranked log2FC values. The significance thresholds were set as a false discovery rate (FDR) q-value < 0.25 and |normalized enrichment score (NES)| > 1.0. Particular attention was given to pathways such as hemoglobin metabolism, iron transport, oxidative stress, and neutrophil degranulation.

### Co-expression network analysis

Differentially expressed lncRNAs and mRNAs obtained by sequencing were used to construct a lncRNA - mRNA co - expression network, aimed at screening for potential lncRNA targets. Center nodes included all significant DE - lncRNAs, while peripheral nodes comprised DE - mRNAs from GSEA - enriched iron metabolism or inflammation pathways (FDR q-value <0.25). Pairwise Pearson correlations (TPM - normalized) with |r| ≥ 0.4 and P-value <0.05 were chosen as the threshold. The networks were constructed and visualized in R using igraph and ggraph, with light blue representing lncRNAs, salmon representing mRNAs, blue edges indicating positive correlations, and red edges indicating negative correlations. Each transcript corresponded to a node, and the lines between nodes represented a strong correlation between them.

### Statistical analysis

Statistical analysis was performed using R version 4.4.1. Data processing, differential analysis, visualization, and functional enrichment analysis were conducted using R packages such as DESeq2, ggplot2, pheatmap, and karyoploteR. Differentially expressed genes were selected based on the criteria of P < 0.05 and |log2 fold change| ≥ 0.5. The network plot revealed gene interactions and their roles in biological processes, and was used to explore key genes associated with the disease. The Benjamini–Hochberg method was used to control the False Discovery Rate (FDR) and adjust the raw p-values for multiple hypothesis testing. The significance threshold was set at an adjusted p-value (FDR) < 0.05.

## Results

### Clinical characteristics

A total of 8 participants were enrolled in the study, comprising 5 patients with type 2 diabetic foot and 3 healthy controls. The mean age of the control group was 69.67 ± 11.85 years, while the mean age of the case group was 58.17 ± 6.40 years. Detailed demographic information for all participants is provided in Table 1.

**TABLE 1:** Comparison of clinical characteristics between the two groups.

Variable	Level	Group A	Group B	P Value
n		3	5	
Gender (%)	Female	1 (33.33)	2 (66.67)	0.635
	Male	2 (40)	3 (60)	
Age (years)		69.67 (11.85)	58.17 (6.40)	0.092
BMI (kg/m2)		21.89 (3.22)	22.13 (2.58)	0.906
DM course (years)		-	8.33 (2.58)	
SBP (mmHg)		136.83 (10.26)	144.83 (23.85)	0.583
DBP (mmHg)		74 (96.10)	79.83 (13.30)	0.954
Fib (g/L)		3.27 (0.64)	4.43 (1.62)	0.280
D-dimer (ug/mL)		2.47 (3.71)	3.41 (3.37)	0.712
TG (mmol/L)		0.84 (0.22)	1.42 (0.48)	0.092
CHO (mmol/L)		5.10 (1.21)	3.47 (1.27)	0.109
HbA1C (%)		-	8.47 (2.45)	
WBC (10^9^/L)		7.66 (0.91)	9.18 (4.56)	0.598
NE (10^9^/L)		5.67 (1.33)	6.62 (3.77)	0.693
HGB (g/L)		132.3 3 (1.53)	93.50 (15.78)	**0.005**
CRP		31.52 (37.49)	51.73 (54.60)	0.6062
Wagner classification		-	4.40 (0.55)	

n, Number of patients; BMI, body mass index; DM, diabetes mellitus; SBP, systolic blood pressure; DBP, diastolic blood pressure; Fib, Fibrinogen; TG, triglycerides; CHO, cholesterol; HbA1C, hemoglobin; WBC, white blood cell count; NE, neutrophil count; HGB, hemoglobin; CRP, C-Reactive Protein; Unpaired t-test (two sides), was used in two group measurement data. Fisher’s exact test was used in enumeration data.Bold values indicate statistical significance at *p* < 0.05.

### Principal component analysis (PCA)

**FIGURE 1:**
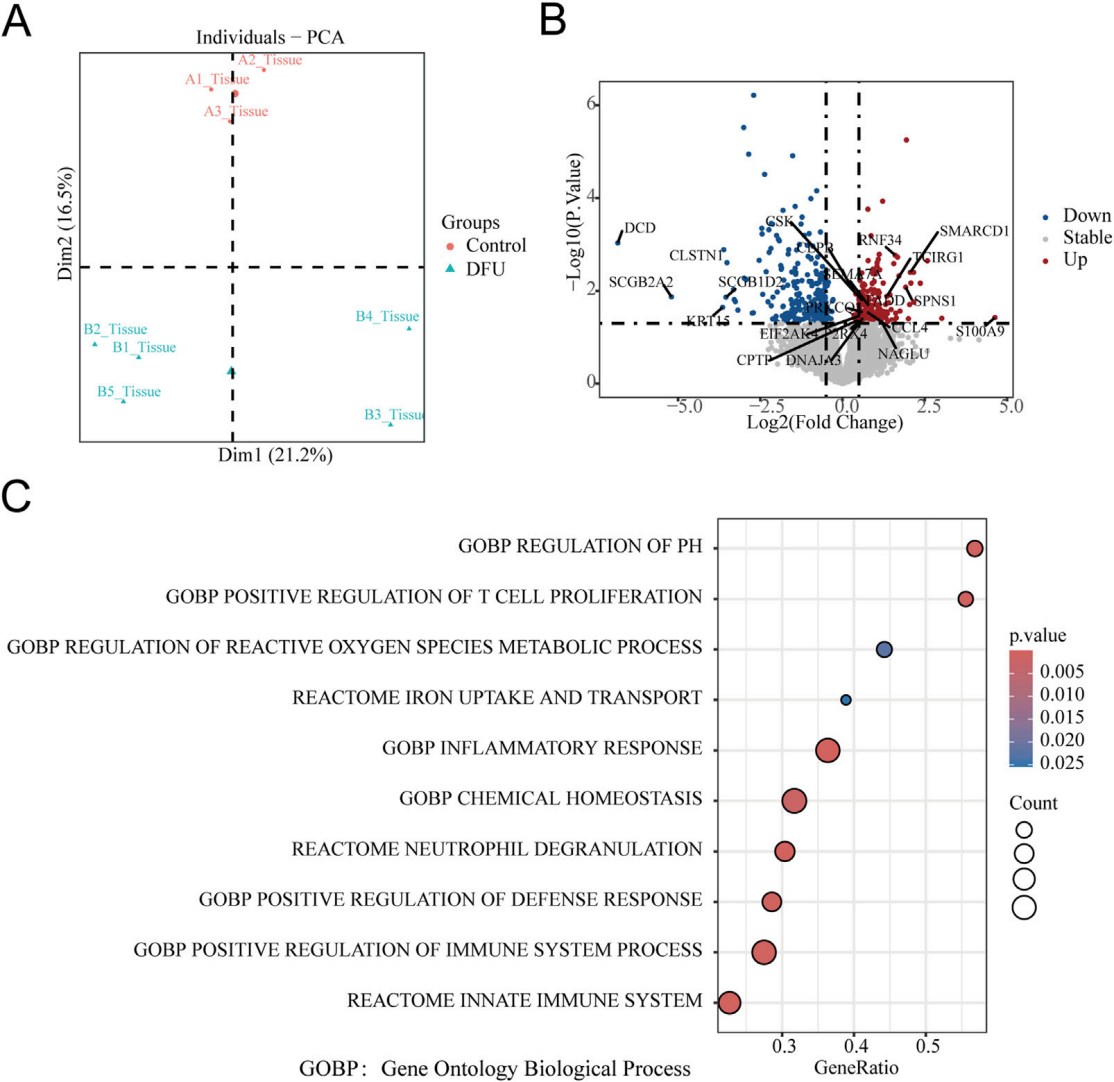
Transcriptomic profiling reveals iron-inflammatory dysregulation in DFU. **(A)** Principal Component Analysis (PCA): Clear separation between control (red) and DFU (green) groups along principal component 1 (Dim1, explaining 16.5%–21.2% variance), indicating distinct transcriptomic signatures. Each point represents an individual tissue sample. **(B)** mRNA Differential Expression Volcano Plot: Significantly downregulated epithelial/neural genes (DCD, CLSTN1, SCGB2A2, KRT15) and upregulated inflammatory/proteolytic genes (SMARCD1, S100A9, RNF34) in DFU vs. controls. Threshold: |log_2_ (fold change)| ≥ 0.5 and FDR < 0.05 (gray: non-significant). **(C)** Bubble Plot of Enriched Pathways: GSEA showing co-enrichment of iron-related and inflammatory pathways. Key pathways: X-axis: Gene ratio (proportion of genes in pathway). Y-axis: Statistical significance (P-value). Bubble size: Number of genes per term (Count). Color intensity: −log_10_(P-value) (darker = more significant).

PCA of skin transcriptomes revealed a clear separation between diabetic foot ulcer (DFU; *n =* 5, red labels: B1-B5) and non-diabetic control (*n =* 3, blue labels: A1, A3) groups along the primary principal component (Dim1, accounting for 21.2% variance), indicating significant global transcriptomic alterations in DFU tissues (Figure 1A).

### Differentially expressed genes

**FIGURE 2:**
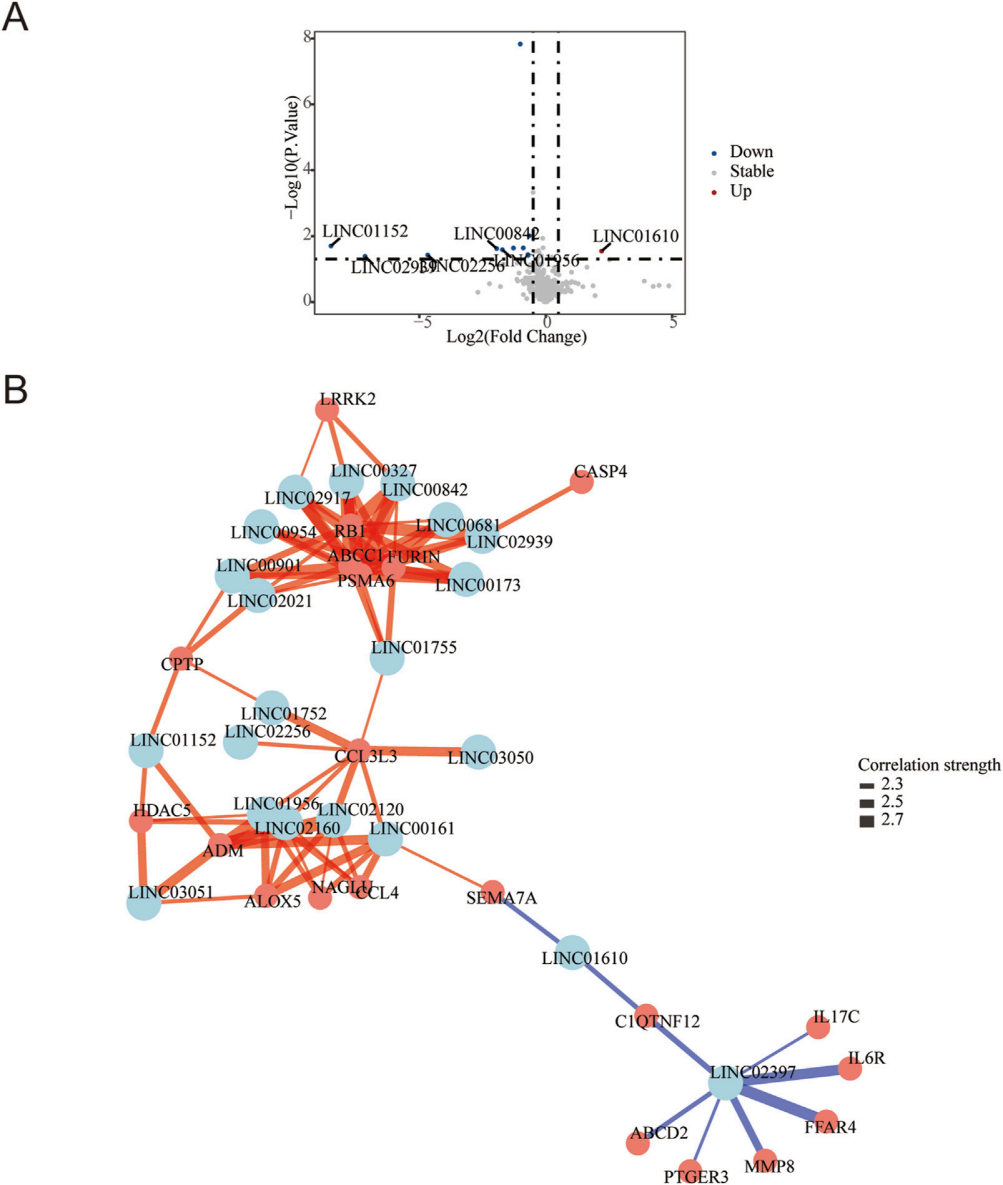
LncRNA Dysregulation and Iron-Inflammatory Gene Network in DFU with Anemia. **(A)** Volcano plot of differentially expressed lncRNAs: Significantly upregulated (red; e.g., LINCO1610) and downregulated (blue; e.g., LINCO1152, LINCO2989) lncRNAs in DFU vs. controls (threshold: |log_2_FC| ≥ 0.5, P < 0.05 by -log_10_(P-value)). Gray points: non-significant lncRNAs. **(B)** Correlation network of lncRNAs with iron/inflammation pathway genes: Central nodes (light blue): Key lncRNAs (p < 0.05). Peripheral nodes (salmon): Iron metabolism/inflammation-related mRNAs. Edges: Blue = positive correlation (r ≥ 0.4), Red = negative correlation (r ≤ −0.4).All connections: |r| ≥ 0.4 and P < 0.05 after pathway-based filtering.

Differential gene expression analysis was conducted using DESeq2, and the results were visualized via a volcano plot. A total of 368 mRNAs were identified as differentially expressed (|log2 FC| ≥ 0.5, P < 0.05), including 139 upregulated genes (e.g., KRT14, CST4, S100A9) and 229 downregulated genes (e.g., SCGB2A2, CLSTN, EDEM3) in DFU compared to controls. These key annotated genes highlight perturbations in critical biological processes such as epidermal barrier function (KRT14), inflammation (S100A9), and stress response (EDEM3) (Figure 1B). Furthermore, 21 lncRNAs were found to be significantly dysregulated in DFU compared to non-diabetic controls, with 20 lncRNAs downregulated and only 1 lncRNA upregulated (LINC01610) (Figure 2A).

### Enriched pathways in DFU

Gene Set Enrichment Analysis (GSEA) using Reactome and Gene Ontology Biological Process (GOBP) gene sets revealed significant upregulation of iron-inflammatory pathways in DFU tissues (FDR q < 0.25, |NES| > 1.0). The most enriched pathway was Reactome: Iron uptake and transport (P < 0.005), indicating profound iron dysregulation. Concurrently, inflammatory and immune activation pathways were upregulated, including Reactome: Neutrophil degranulation, GOBP: Inflammatory response, and Reactome: Innate immune system. Oxidative stress processes (GOBP: Regulation of reactive oxygen species metabolic process) and immune cell proliferation (GOBP: Positive regulation of T cell proliferation) were also significantly elevated (Figure 1C).

### Co-expression network reveals lncRNA-mRNA regulatory hubs in DFU

The targeted co-expression network integrated 21 differentially expressed lncRNAs (center nodes, light blue) with iron metabolism and inflammation-associated mRNAs (peripheral nodes, salmon), uncovering critical regulatory architectures in DFU pathogenesis. The primary network hub was identified as LINC02397, which exhibited strong positive correlations (r > 0.7, P < 0.01) with multiple pro-inflammatory mediators: cytokine signaling components (IL6R, IL17C), tissue remodeling enzyme MMP8, metabolic sensors (FFAR4, ABCD2), and inflammatory mediator PTGER3. Concurrently, suppressive lncRNA-mRNA axes emerged, most notably with LINC01610 showing significant negative correlations (r < −0.6, P < 0.05) to immunomodulators SEMA7A and CIQTNF12 (Figure 2B).

## Discussion

Anemia is prevalent in diabetes mellitus, particularly among patients with diabetic foot ulcers (DFU), potentially impeding healing and increasing amputation/mortality risks due to impaired microcirculation. A meta-analysis of 15 studies (*n =* 2,895 patients) revealed weighted anemia prevalences of 69.7% (total DFU), 49.5% (mild-to-moderate DFU), and 73.0% (severe DFU) (Yammine et al., 2021). Our transcriptomic analysis provides new insights into the pathogenesis of DFU-associated anemia, highlighting lncRNAs as master regulators of an iron-inflammatory-metabolic axis. Unlike prior models that primarily emphasize hyperglycemia-induced vascular damage or nutritional deficiencies, our findings uncover a previously unrecognized lncRNA-mediated regulatory architecture that drives iron-inflammatory dysregulation in DFU tissues from patients with concomitant anemia.

The significant enrichment of Reactome: Iron uptake and transport alongside inflammatory pathways (Neutrophil degranulation, Inflammatory response) establishes a molecular framework for iron-inflammatory dysregulation in diabetic foot ulcers with hemoglobin deficiency. Our clinical data confirm a significant reduction in hemoglobin levels in diabetic foot ulcer (DFU) patients compared to non-diabetic controls, consistent with the established prevalence of anemia in chronic wounds (Li J. et al., 2023). Transcriptomically, profound downregulation of epithelial defense genes—including secretoglobin family members SCGB2A2, which modulates IL-1β signaling; SCGB1D2, an anti-protease inhibiting neutrophil elastase; and DCD, an antimicrobial peptide critical for skin barrier function—reflects multilayered barrier compromise (Onida et al., 2025). This suppression facilitates bacterial invasion and unchecked proteolytic activity, as evidenced by MMP8 upregulation in our co-expression network (Li S. et al., 2023). Resultant TLR4-driven inflammation propagates IL-6 overproduction through LINC02397-mediated IL6R activation, directly suppressing erythropoiesis and impairing hemoglobin synthesis (Rui et al., 2024). Concurrent neural-epithelial disruption arises from suppression of CLSTN1, essential for sensory nerve communication, and KRT15, maintaining epidermal stemness. These deficits collectively permit uncontrolled IL17C activation (r = 0.718 with LINC02397) and neutrophil-mediated tissue destruction, creating a self-reinforcing cycle of barrier failure, chronic inflammation, erythropoietic suppression, and impaired hemoglobin production that perpetuates DFU progression (Percival et al., 2012; Louiselle et al., 2021; Liu et al., 2025).

The profound transcriptional imbalance in diabetic foot ulcers with anemia—characterized by suppression of 20 lncRNAs regulating epithelial integrity (LINC01752, LINC03050), neural function (LINC00173, LINC01152), and inflammation control (LINC00327, LINC02939), alongside exclusive upregulation of LINC01610—reflects systemic failure of regulatory networks governing tissue homeostasis. Mechanistically, barrier collapse initiates this pathology: co-downregulation of LINC01752 correlates with DCD deficiency, impairing antimicrobial defense; LINC03050 targets the SCGB2A2 promoter, disabling anti-protease activity; and LINC02939 destabilizes KRT15 mRNA, depleting epidermal stem cells. This barrier breach facilitates bacterial invasion, triggering TLR4/MMP8-driven inflammation that induces hepcidin-mediated iron blockade (Shi et al., 2023). Concurrently, LINC01610 upregulation disrupts neuroimmune crosstalk through positive correlation with SEMA7A—amplifying neuropathic pain—and negative regulation of CLSTN1, compromising neuron-keratinocyte communication. These defects collectively disable neurogenic inflammation resolution, perpetuating IL-17 signaling. Critically, suppressed anti-inflammatory lncRNAs exacerbate this cascade: LINC00327 normally targets IL6R 3′UTR to constrain signaling, while LINC02397 binds NF-κB repressors; their loss enables constitutive IL-6/NF-κB activation that promotes hepcidin overexpression and macrophage iron sequestration, completing a vicious cycle of anemia and non-healing (Li et al., 2022; Anguiano-Hernandez et al., 2019).

As the highest-degree node within the co-expression network, LINC02397 emerges as a master regulator of cytokine-driven erythropoietic suppression through its robust positive correlations with core inflammatory effectors. IL6R, whose activation sustains JAK-STAT3 signaling to induce hepcidin—a key hormone that blocks duodenal iron absorption, as established in anemia of inflammation (Yadav et al., 2024). IL17C, where overexpression promotes neutrophil infiltration (Mobile et al., 2023), triggering reactive oxygen species release that directly damages erythrocytes. PTGER3, whose signaling escalates vascular permeability (Morimoto et al., 2014), exacerbating ulcer hypoxia and impairing erythrocyte production. This triad of LINC02397-driven inflammatory dysregulation—iron malabsorption, oxidative erythrocyte damage, and microvascular dysfunction—collectively explains the therapeutic resistance of DFU-associated anemia to iron supplementation, positioning unmitigated inflammation rather than absolute iron deficiency as the primary pathological driver.

### Limitations

Our network analysis provides foundational insights into lncRNA-mediated iron-inflammatory dysregulation in DFU-anemia, yet future research must address key gaps: cohort expansion to ensure robustness, functional validation of regulatory mechanisms, resolution of cell-type-specific functions, and integration of dynamic hematological parameters. Only through such multidimensional validation can these findings mature into targeted therapies.

## Conclusion

In DFU with anemia, coordinated suppression of 20 lncRNAs disables barrier protection (DCD/SCGB2A2), neural repair (CLSTN1), and inflammation control (IL6R), while upregulation of LINC01610 hijacks neuroimmune signaling (SEMA7A). This dual dysregulation creates a self-sustaining ecosystem where iron becomes biologically inaccessible. Restoring key lncRNAs (e.g., LINC01752, LINC00327) and silencing LINC01610 offers a precision medicine approach to reverse anemia and heal wounds.

## Data Availability

The data presented in the study are deposited in the NCBI repository, accession number PRJNA1234057.
